# Prevalence of Sleep Disorders and Their Impacts on Occupational Performance: A Comparison between Shift Workers and Nonshift Workers

**DOI:** 10.1155/2014/870320

**Published:** 2014-05-20

**Authors:** Zohreh Yazdi, Khosro Sadeghniiat-Haghighi, Ziba Loukzadeh, Khadijeh Elmizadeh, Mahnaz Abbasi

**Affiliations:** ^1^Metabolic Disease Research Center, Qazvin University of Medical Sciences, Qazvin 34139-8-3731, Iran; ^2^Occupational Sleep Research Center, Tehran University of Medical Sciences, Tehran 5583-1-4155, Iran; ^3^Department of Occupational Medicine, Industrial Diseases Research Center, Shahid Sadoughi University of Medical Sciences, Yazd 89151-7-3143, Iran; ^4^Social Determinants of Health Research Center, Qazvin University of Medical Sciences, Qazvin, Qazvin 34139-8-3731, Iran

## Abstract

The consequences of sleep deprivation and sleepiness have been noted as the most important health problem in our modern society among shift workers. The objective of this study was to investigate the prevalence of sleep disorders and their possible effects on work performance in two groups of Iranian shift workers and nonshift workers. This study was designed as a cross-sectional study. The data were collected by PSQI, Berlin questionnaire, Epworth Sleepiness Scale, Insomnia Severity Index, and RLS Questionnaire. Occupational impact of different sleep disorders was detected by Occupational Impact of Sleep Disorder questionnaire. These questionnaires were filled in by 210 shift workers and 204 nonshift workers. There was no significant difference in the age, BMI, marital status, and years of employment in the two groups. Shift workers scored significantly higher in the OISD. The prevalence of insomnia, poor sleep quality, and daytime sleepiness was significantly higher in shift workers. Correlations between OISD scores and insomnia, sleep quality, and daytime sleepiness were significant. We concluded that sleep disorders should receive more attention as a robust indicator of work limitation.

## 1. Introduction

The consequences of sleep deprivation and sleepiness have been noted as the most important health problem in our modern society among shift workers. These consequences include increase in mortality, morbidity, accidents and errors, absenteeism in workplace, decrease in productivity, and deterioration of personal and professional relationships [1–3].

The prevalence of sleep disorders is common in shift workers but is often underdiagnosed [4]. The association between sleep disorders and shift work is bidirectional. Shift work induces some sleep complaints such as insomnia, poor sleep quality, and daytime sleepiness. On the other hand, underlying sleep disorders decrease workers' capabilities to adapt with shift working and increase accidents at work [5, 6].

Insomnia is the most prevalent sleep disorder among the adults. The estimated prevalence of difficulty in initiating and maintaining sleep is about 30% [7]. In spite of this reality, relationship between insomnia and work performance has received little attention in researches [8].

It is estimated that 4% of males and 2% of females suffer from obstructive sleep apnea (OSA) and the majority of patients are thought to be undiagnosed. Certainly, investigators have shown that OSA is associated with large increase in healthcare costs in working age adults [9, 10]. However, few studies have specifically surveyed the impact of undiagnosed OSA and resultant sleepiness on work disabilities such as work absences and decreased productivity [11]. A recent study has reported that the combination of OSA and resultant excessive daytime sleepiness contributes to work disability in patients who underwent overnight polysomnography [12].

There are few studies regarding work disabilities due to other sleep disorders such as restless legs syndrome, periodic limb movement syndrome, and parasomnia. But theoretically, all of these sleep disorders are generally associated with either sleep fragmentation or decreased sleep quality [13].

Sleep quality is an important clinical construct for being healthy people. Poor sleep quality can be an important symptom of many sleep disorders and other medical diseases and might even have a direct effect on increased mortality [14].

Despite the high prevalence of sleep disorders, there are few studies on their effects on work limitations. Verster et al. assessed the validity of the Occupational Impact of Sleep Questionnaire in Dutch language. According to their results Dutch version OISD was a suitable instrument to estimate the occupational impact of sleep disorders. Also, they showed that poor sleepers had higher scores of OISD compared to good sleepers [8].

The aim of this study was to evaluate the prevalence of sleep disorders and their possible effects on work performance in two groups of Iranian shift workers and nonshift workers.

## 2. Materials and Methodology

A self-administered questionnaire was submitted to all workers employed in textile factory. A total of 225 shift workers and 245 nonshift workers participated in this study. All the participants were informed about the objectives of the study and the methods used during the survey. Each participant received the questionnaire and completed it during the work hours.

The Persian version of the questionnaires was used to detect the sleep disorders in workers. Insomnia Severity Index, Berlin questionnaire, Epworth Sleepiness Scale, and Pittsburg Sleep Quality Index were used to screen insomnia, obstructive sleep apnea, daytime sleepiness, and sleep quality, respectively [15–17]. Berlin questionnaire consists of three categories. The first, second, and third categories have questions about snoring, daytime somnolence, and existence of high blood pressure, respectively. The patient is considered high risk for OSA if two or more categories are positive [18].

We used the four minimal IRLSSG clinical criteria for screening of RLS in participants. All cases who responded positively to the first three questions of questionnaire were considered to have RLS [19].

We used the Occupational Impact of Sleep Disorder (OISD) questionnaire to detect the occupational impact of sleep disorders. This questionnaire included 23 items, and each item was organized into four levels as follows: “Never,” “Rarely,” “Sometimes,” and “Often,” with a numeric value of 0, 1, 2, and 3, respectively, and a total score from 0 to 96 [8]. At first, we translated the OISD questionnaire by bilingual expert panel of sleep physicians and occupational medicine specialist. In the translation process, we choose an easily understandable style and a wording with the same meanings of original questionnaire. Then we back-translated into English by another two translators, who did not participate in any of the previous steps. At last, individuals who were fluent in English compared original OISD with the final back-translated form. The OISD internal consistency in our study was high with a Cronbach's alpha of 0.83.

SPSS for windows version 13 was used for statistical analysis. Summary statistics for descriptive data were obtained for means and standard deviations. We used Cronbach's alpha to test internal consistency reliabilities and Pearson correlation coefficients to compute correlations among the measures. Student's *t*-tests and chi-square analyses were performed to look for group differences. For all data, a *P* value of <0.05 was considered statistically significant.

## 3. Results

A total of 470 questionnaires were completed including 210 shift workers and 204 nonshift workers. Incompletely filled questionnaires were excluded from the study. All participants were male, and the mean age was 34.8 ranging from 22 to 45.  Table 1 shows sociodemographic data of participants. There were no significant differences in the age, BMI, marital status, and years of employment.

Shift workers reported lower education compared with nonshift workers and scored significantly higher in the OISD, indicating they tolerated more occupational impacts of sleep disorders than nonshift workers (27.2 versus 16.1; *P* = 0.001).

The prevalence of insomnia, poor sleep quality, and daytime sleepiness was significantly higher in shift workers than nonshift workers (Table 2).

Table 3 shows that the correlations between OISD scores and insomnia, sleep quality, and daytime sleepiness are high and significant. Correlations are stronger in the shift workers group. There is not any correlation between OISD scores and RLS and sleep apnea in both groups.

## 4. Discussion 

This study evaluated the impacts of sleep disorders on occupational performance in an industrial setting. The major finding of this study was a significant increase in sleep disorders among the shift workers than in nonshift workers. In addition, occupational impacts of these sleep disorders were more profound in this group. Despite the high prevalence of different sleep disorders in the general population, there is a lack of studies on the harmful effects of these disorders on job performance [8]. Overall, the result of our study is consistent with limited studies performed in this field. In a study about OSA that was conducted in patients admitted to a sleep clinic, it was found that patients with excessive daytime sleepiness (assessed by the Epworth Sleepiness Scale) were more likely to suffer from work limitation in time management, work efficiency, and mental interpersonal relationship. They did not find such correlation in OSA patients without daytime sleepiness [12].

In another study it was found that excessive daytime sleepiness is related to increased rate of stress and interpersonal tensions at work [11]. In addition, several studies have reported improvement in work performance after treatment of OSA patients with excessive daytime sleepiness [10, 20].

In our study, the prevalence of EDS was higher in shift workers than in nonshift workers and in both groups it was accompanied by higher OISD scores. Also, our study showed that there was no difference between the prevalence of OSA in shift workers and nonshift workers on the basis of the Berlin questionnaire. They also found no relation between OSA and the scores that resulted from OISD questionnaire. The absence of this relation can be attributed to the small number of cases detected to have OSA using the Berlin questionnaire.

In many studies, the prevalence of insomnia has been evaluated in general population and shift workers and the results of these studies show that insomnia is more prevalent in shift workers in contrast to general population [4, 14, 21]. In this regard the result of our study is consistent with other studies. As the results of our study show, among the sleep disorders, insomnia and low sleep quality had the highest correlation with the incidence of negative occupational impacts. As impaired daily functioning is one of the diagnostic items for detecting sleep disorders, the above correlation is completely logical. In another study using sleep50 questionnaire for evaluation of sleep disorders, researchers showed powerful association between insomnia negative occupational impacts [8].

Other studies have also shown the same results. Previous studies have revealed that it is twice more probable for workers with insomnia to lose their work. They have lower self-confidence and occupational satisfaction and lower efficacy in work. In addition, patients with insomnia disorder are 3 times more prone to dangerous road accidents. The studies have also shown that these workers have 1.4 times more absence from work than the workers without insomnia [22–26]. It seems that, with the use of the OISD questionnaire, it is possible to prevent these complications at the first stages. On the other hand, it is obvious that absence from work is the end point of the problems in waking up on time to work and on time arrival and the ability to work the whole work time. Thus, assessment of the above problems in questionnaire is very important and necessary for occupational investigations.

Another considerable result of the present study is more prevalence of poor sleep quality in the shift workers in contrast to nonshift workers. In both groups, poor sleep quality culminated in negative impacts on work performance. Of course this impact was stronger in shift workers than the others. In one study performed in Denmark there was a meaningful association between the OISD questionnaire and the quality of sleep [8].

It is necessary to say that the relation between the quality of sleep and occupational performance is two-sided. On one hand, low quality of sleep can result in poor work performance and work accidents. On the other hand, problems occurring while working can culminate in sleep disorders [25]. Knudsen et al. in their study showed that the workers who had work accidents resulting in work absence in the recent past year were more prone to insomnia disorder and poor sleep quality [26].

Different factors can affect sleep quality including personal characteristics sleep biology, circadian rhythm, and shifting work [27]. Our study revealed that poor quality of sleep can be seen in both shift workers and nonshift workers with a meaningful higher prevalence in the former. Different studies have reported higher incidence of tension, depression, and tiredness in shift workers. They are not spiteful when awaking and are more tired along the day. This is due to discrepancy between circadian rhythm and sleeping and awaking time in these populations [28, 29].

In the present study, we found no statistical difference in the prevalence of RLS among the shift workers and nonshift workers. Also, there was no statistical correlation between the scores from OISD questionnaire and the presence of RLS. In this regard, our results are different from the results of another study that evaluated the prevalence of RSL in shift and nonshift workers [30]. The present discrepancy can be due to small number of cases complaining about RSL in our study.

In the study, we showed the scores from OISD questionnaire to be significantly higher in shift workers than in nonshift workers. In some studies that used other questionnaires to assess the work ability index, they showed that this index was different among shift and nonshift workers both in males and females [31]. In different studies, different questionnaires have been designed to evaluate the occupational impact and work limitations due to sleep disorders in various populations. In most of these studies the effects of OSA on work limitation have been evaluated [11, 12, 32].

We used the related standard questionnaire to diagnose each sleep disorder and this is a positive point of our study. As a point of weakness, we evaluated various sleep disorders only subjectively and we did not use objective tests such as polysomnography. The other limiting factor of the paper is the fact that all the participants in the study are male, so we cannot extrapolate the results to the female shift workers.

It is suggested that in future studies the result of questionnaires be evaluated in different groups of sleep disorder proved on the basis of subjective methods. It is also suggested to investigate the effect of treatment on the scores of patients. However, in further studies on OISD the effect of different variables including age, sex, and various personality characteristics such as preferred sleep time can be evaluated. Furthermore, for the validity assessment of the questionnaire, the association between the questionnaire results and the incidence of work errors and accidents can be investigated.

Referring to our study results, OISD questionnaire is a suitable tool for assessment of the effect of sleep disorders on work ability. We propose that the efficacy of this questionnaire be evaluated in different groups of workers like HCWs, professional drivers, pilots,workers who spent long hours in front of a computer screen, and overall workers with heavy work load.

## Supplementary Material

Appendix 1: Occupational Impact of Sleep Questionnaire (OISQ)
Quality of sleep can influence our ability to perform in the workplace. The following questions relate to ways in which your work performance may have been affected by your sleep during the past week. Please Indicate (×) how often each item applied to you. Answer all the questions.

## Figures and Tables

**Table 1:** Baseline characteristics in two groups of shift workers and nonshift workers.

Characteristics	Shift workers *N* = 210	Nonshift workers *N* = 204	*P* value
Age	33.7 ± 5.9	35.1 ± 10.9	0.1
BMI	22.8 ± 3.1	23.5 ± 4.4	0.08
Marital status (married)	173 (%)	159 (%)	0.31
Educational status	9.3 ± 3.6	12.9 ± 5.1	0.04
Years of employment	12.1 ± 8.5	13.7 ± 10.3	0.086
OISD	27.2 ± 9.8	16.1 ± 10.3	0.001

**Table 2:** Prevalence of different types of sleep disorders in two groups of shift workers and nonshift workers.

		Shift workers	Nonshift workers	*P* value
Insomnia	Yes	26 (12.4%)	14 (6.8%)	*P* = 0.02
	No	184 (87.6%)	190 (93.1%)	

Sleep quality	Poor sleep quality	43 (20.4%)	21 (10.3%)	*P* = 0.004
	Better sleep quality	167 (79.5%)	183 (89.7%)	

ESS	With ESS	21 (10%)	13 (6.4%)	*P* = 0.041
	Without ESS	189 (90%)	191 (93.6%)	

RLS	Yes	22 (10.5%)	14 (6.8%)	*P* > 0.05
	No	188 (89.5%)	190 (93.1%)	

Sleep apnea	Yes	15 (7.1%)	17 (8.3%)	*P* > 0.05
	No	195 (92.8%)	187 (91.6%)	

**Table 3:** Correlations between different types of sleep disorders with OISD in two groups of shift workers and nonshift workers.

	Insomnia	Sleep quality	ESS	RLS	Sleep apnea
OISD in shift workers	0.52*	0.48*	0.39**	0.14	0.04
OISD in nonshift workers	0.37*	0.36*	0.21*	0.06	0.11

*P < 0.05; **P < 0.01.

## Abbreviations

PSQI: Pittsburg Sleep Quality Index
ESS: Epworth Sleepiness Scale
ISS: Insomnia Severity Index
OISD: Occupational Impact of Sleep Disorder questionnaire.
