# Supplementary material for: Prevalence of Sleep Disorders and Their Impacts on Occupational Performance: A Comparison between Shift Workers and Nonshift Workers
Source: Sleep Disord. 2014 May 20;2014:870320. doi: 10.1155/2014/870320 (PMC4055012; doi:10.1155/2014/870320)
Supplement: Supplementary file 1 — Appendix 1: Occupational Impact of Sleep Questionnaire (OISQ) Quality of sleep can influence our ability to perform in the workplace. The following questions relate to ways in which your work performance may have been affected by your sleep during the past week. Please Indicate (×) how often each item applied to you. Answer all the questions. [file 870320.f1.docx]

**Appendix 1: OCCUPATIONAL IMPACT OF SLEEP QUESTIONNAIRE (OISQ)**

Quality of sleep can influence our ability to perform in the workplace. The following questions relate to ways in which your work performance may have been affected by your sleep during the past week. Please Indicate (×) how often each item applied to you. Answer all the questions.

During the past week, how often did the quality of your sleep make it difficult for you to:

|  |  | All of the time | Most of the time | Some of the time | A little bit of the time | Never/Not Applicable |
| --- | --- | --- | --- | --- | --- | --- |
| 1 | Wake up for work on time? |  |  |  |  |  |
| 2 | Arrive at work on time? |  |  |  |  |  |
| 3 | Work the required number of hours? |  |  |  |  |  |
| 4 | Get going easily at the beginning of the workday? |  |  |  |  |  |
| 5 | Start on your job as soon as you arrive at work? |  |  |  |  |  |
| 6 | Do your work without stopping to take breaks or rests? |  |  |  |  |  |
| 7 | Keep working effectively during the afternoon? |  |  |  |  |  |
| 8 | Maintain your stamina throughout the day? |  |  |  |  |  |
| 9 | Keep to a routine or schedule? |  |  |  |  |  |
| 10 | Think clearly when working? |  |  |  |  |  |
| 11 | Keep your mind on your work? |  |  |  |  |  |
| 12 | Do work carefully? |  |  |  |  |  |
| 13 | Concentrate on your work? |  |  |  |  |  |
| 14 | Work without losing your train of thought? |  |  |  |  |  |
| 15 | Easily read or use your eyes when working? |  |  |  |  |  |
| 16 | Speak with people in-person, in meetings or on the phone? |  |  |  |  |  |
| 17 | Control your temper around people when working? |  |  |  |  |  |
| 18 | Help other people to get work done? |  |  |  |  |  |
| 19 | Handle the workload? |  |  |  |  |  |
| 20 | Work fast enough? |  |  |  |  |  |
| 21 | Finish work on time? |  |  |  |  |  |
| 22 | Do your work without making mistakes? |  |  |  |  |  |
| 23 | Feel you have done what you are capable of doing? |  |  |  |  |  |

Scoring: All of the time (0), Most of the time (1), Some of the time (2), A little bit of the time (3), Never/Not applicable (4).

Adapted from: Verster JC, David B, Morgan K, Olivier B. Validation of the Dutch Occupational Impact of Sleep Questionnaire (OISD). Industrial Health 2008; 46: 601-606.
